# Impact of Biochar on Rhizosphere Bacterial Diversity Restoration Following Chloropicrin Fumigation of Planted Soil

**DOI:** 10.3390/ijerph19042126

**Published:** 2022-02-14

**Authors:** Jun Li, Yan Chen, Xiangyang Qin, Aocheng Cao, Anxiang Lu

**Affiliations:** 1 Institute of Quality Standard and Testing Technology, Beijing Academy of Agriculture and Forestry Science, Beijing 100097, China; lij@brcast.org.cn (J.L.); qinxiangyang@baafs.net.cn (X.Q.); 2 Institute of Plant Protection, Chinese Academy of Agricultural Sciences, Beijing 100193, China; caoac@vip.sina.com; 3 COFCO Nutrition & Health Research Institute, Beijing 102209, China; agroeco2000@outlook.com

**Keywords:** beneficial microorganisms, biochar amendment, chloropicrin, rhizobacterial diversity, soil fumigation

## Abstract

Chloropicrin (CP) can effectively combat soil-borne diseases but has significant side effects on nontarget microorganisms. The rhizosphere microflora play a crucial role in promoting plant growth and protecting plants from infection by soil-borne pathogens. We conducted a laboratory pot experiment to evaluate the effect of CP on the rhizosphere soil bacterial flora and the effect of biochar amendments on the reconstruction of microbial communities. Our results show that CP fumigation and biochar additions promoted the growth of cucumber plants in the later stage of the pot experiment. CP significantly inhibited the rhizobacterial diversity and changed the community composition. Biochar amendments after CP fumigation shortened the time for the rhizobacterial diversity to recover to unfumigated levels. Biochar amendments promoted the transplantation of new populations to empty microbiome niches that were caused by CP and, in particular, stimulated many beneficial microorganisms to become the predominant flora. The relative abundances of many functional taxa related to plant-disease suppressiveness and pollutant bioremediation increased, including *Pseudomonas*, *Stenotrophomonas*, *Bacillus*, *Massilia*, *Acinetobacter*, *Delftia*, *Micromonospora*, Cytophagaceae, and *Flavisolibacter*. These changes stimulated by biochar amendments would promote multifunctionality in the soil rhizosphere and benefit plant growth and disease resistance.

## 1. Introduction

Soil fumigation is recognized as an effective method to combat soil-borne diseases. As a fumigant with the broadest range of applications, methyl bromide (MB) has an excellent control effect on soil-borne pathogens, pests, and weeds [1]. However, under the provisions of the Montreal Protocol, developing countries were required to phase out controlled uses of MB by 1 January 2015 because of its ozone-depleting properties [2]. As one of the substitutes for MB, chloropicrin (CP) can provide broad-spectrum and effective control of soil-borne pathogenic bacteria, fungi, nematodes, and weeds, such as *Ralstonia solanacarum*, *Verticillium dahlia*, *Fusarium oxysporum*, *Meloidogyne*, and *Cyperus rotundus* L. [3,4]. In China, approximately 10,000 tons of CP are produced every year for pre-plant soil fumigation (official data from the Ministry of Agriculture and Rural Affairs of China in 2017).

The soil microflora are crucial components in the pedosphere, and they drive and maintain many essential processes in soil ecosystems, which include the promotion of plant growth, biogeochemical cycling of plant nutrient elements, and degradation of pollutants [5,6]. The application of CP will cause external disturbances to the diversity and composition of the soil microflora, which may affect the functional potentials of soil ecosystems and cause ecological risks [7,8]. The specific impacts and durations depend on the characteristics of the fumigants and types of soils. Li et al. [7,9] found that applications of different fumigants caused varying degrees of change in soil bacterial communities, including decreases in diversity, shifts of the structural composition of soil bacterial communities, and inhibition of the expressions of some essential genes in the nitrogen cycling process. Fang et al. [10] found that soil fumigation significantly changed the abundances and community structure of denitrifying bacteria. However, most of these studies used laboratory simulation culture methods, which only focused on the bulk soil microbial communities, and less attention was given to changes of rhizosphere soil microflora in the soil ecosystems after the application of fumigants.

Plant rhizosphere soil microbiomes play a vital role in plant nutritional acquisition from the soil and benefit plants by preventing infection from soil-borne pathogens [11]. Therefore, research on rhizosphere microflora is crucial for a better understanding of the interactions between crop production and microorganisms in agricultural soil ecosystems that are treated with fumigants. Previous studies have used PCR-Denaturing Gradient Gel Electrophoresis (DGGE) to detect the effects of fumigants on plant rhizosphere microorganisms [12,13]. In recent years, high-throughput sequencing for microbial communities has widely been used to investigate the response of the rhizosphere microbiome to fumigants, and more details about the changes in microbial composition have also been discovered [14].

As a soil amendment agent, biochar has been described as a possible substance to improve soil fertility and mitigate climate change [15]. Previous studies have shown that biochar amendments after soil fumigation can significantly reduce the emissions of fumigants into the atmosphere [16]. Liu et al. [17] found that biochar can accelerate the degradation of CP and is beneficial for removing fumigants thoroughly from the soil. Zhang et al. [18] found that biochar increased the richness and diversity of the bacterial community in the tobacco rhizosphere. Sheng and Zhu [19] found that biochar increased the relative abundances of copiotrophic bacteria, such as Bacteroidetes and Gemmatimonadetes. Fang et al. [20] found that the time for the recovery of bacterial diversity in the biochar-amended bulk soil after CP fumigation was 28 days shorter than that in non-biochar-amended soils. These properties of biochar may make it a soil improver following soil fumigation. However, these studies were most often carried out with microscopic methods to investigate the response of microbiota in the bulk soil to biochar amendments and CP fumigation, without taking into account the important and complex effects of crop growing on soil microbial recovery. Few studies have reported changes of rhizobacterial communities after biochar amendments in fumigated soils.

In this study, we focused on the dynamic changes of the rhizosphere microbiome after CP fumigation and the role of biochar in the process of rhizosphere bacterial diversity restoration following CP fumigation of planted soil. Taking into account the differences between the microbiome from bulk soil and the rhizosphere soil, we hypothesized that (1) CP would have similar but not exactly the same effects on the microbial community in the rhizosphere soil as that in the bulk soil and (2) biochar would benefit restoration and reconstruction of the rhizosphere microbial community following CP fumigation. We used laboratory pot experiments combined with high-throughput sequencing technology to monitor the changes in the diversity and structural composition of the bacterial communities in cucumber (*Cucumis sativus* L.) seedlings in rhizosphere soil after CP fumigation and biochar amendments. Here we used cucumbers as potted plants for two reasons: (1) cucumbers are one of the main vegetables in China, and (2) before planting cucumbers, CP is usually used to disinfect the soil to avoid soil-borne diseases such as cucumber fusarium wilt, cucumber phytophthora blight, and cucumber root-knot nematodes.

## 2. Materials and Methods

### 2.1. Preparation of Used Soil in This Study

The soil used for the laboratory experiments was collected from Yanqing District, Beijing, China (GPS coordinates 40°03′24.6″ N, 116°56′14.6″ E). The soil was composed of 5% coarse sand, 64% fine sand, 25% silt, and 6% clay. The soil had a pH of 6.36, NH_4_^+^-N 25.45 mg kg^−1^, NO_3_^−^-N 214.10 mg kg^−1^, available P 692.73 mg kg^−1^, available K 596.67 mg kg^−1^, organic matter 49.48 g kg^−1^, and electrical conductivity of 460.33 μs cm^−1^. The soil was homogenized by sieving (2 mm mesh). The soil moisture was adjusted to 60% of the maximum water-holding capacity (WHC) by using sterile distilled H_2_O, according to the guidelines for CP soil disinfection in China, the China Agricultural Standard, NY T2725-2015.

The soil was fumigated by CP in a plastic box for a pot experiment. The CP (a liquid product containing 99.5% CP, Dalian Dyestuffs & Chemicals Co., Dalian, China) concentrations were set to 70 mg kg^−1^ and 35 mg kg^−1^ as the high concentration treatment and low concentration treatment, respectively (assuming that CP is distributed evenly over a 20 cm soil depth and a soil bulk density of 1.4 g cm^−3^). These application rates are consistent with the recommended application rates in the guidelines for CP soil disinfection in China and are consistent with previous studies [21,22]. The plastic box used for fumigation was covered with polyethylene film (0.03 mm thick), and polyethylene tape was wrapped around the box to ensure sealing. The fumigation lasted for seven days at 25 °C. The boxes were then opened and placed in a fume cupboard for three days to eliminate the residual CP.

### 2.2. Glasshouse Pot Experiments

The pot experiment included five different treatments: unfumigated soil (Non & CK), high-dosage CP fumigated soil (High & CK), low-dosage CP fumigated soil (Low & CK), unfumigated soil with biochar addition (Non & Bio), and high-dosage CP fumigated soil with biochar addition (High & Bio). The biochar used in this study was produced from a weed, *Ageratina adenophora* (Spreng.) R.M.King & H.Rob. The biochar production from air-dried *A. adenophora* was conducted in an O-KTF1200 vacuum tube furnace (Chunlei Co. Ltd., Yancheng, China) under the following conditions: a heating rate of 7 °C min^−1^ up to 500 °C for 2 h and a N_2_ flow of 500 mL min^−1^. The biomass after combustion was ground and then filtered with a 0.2 mm (120 mesh) sieve. The biochar was composed of 68.43% C, 4.01% O, 0.72% H, and 0.93% N, with a specific surface area (SSA) of 162.18 m^−2^·g^−1^, a pH of 10.5, and ash content of 4.5% (*w*/*w*, dry weight basis). Biochars were added after soil fumigation and ventilation, and before cucumber seeds were sown. The addition ratio of biochar was 2% (*w*/*w*).

A sample of soil (2 kg, dw) was placed in a plastic pot (volume 2.46 L, diameter 14 cm, height 16 cm). One pregerminated cucumber (*Cucumis sativus* L.) seed was planted 1.0 cm deep in each pot. All the pot experiments were conducted in a glasshouse at the Chinese Academy of Agricultural Sciences under natural sunlight conditions. The positions of the pots were adjusted every 3 d to avoid the difference caused by uneven natural factors such as lighting. Pots were irrigated as needed. The cucumber plants were sampled from the glasshouse at 7 days, 15 days, 30 days, 45 days, and 60 days. Three seedlings were sampled for each treatment every time.

### 2.3. Sampling and Determination of Plant Growth Responses

After the plants were carefully removed from the pots, the heights and stem thicknesses of the cucumber plants were measured. The parts above the soil layer of the cucumbers were cut off using sterile scissors. Rhizosphere soils were obtained by vortexing roots in a buffer (phosphate-buffered saline, PBS) [23].

The genomic DNA was extracted from the soil samples using a MoBio Power Extraction kit (MoBio, Carlsbad, CA, USA) according to the manufacturer’s instructions. The quality of the extracted DNA was verified using a Nanodrop1000 spectrophotometer (Thermo Fisher Scientific, Hudson, NH, USA) and 0.5% (*w*/*v*) agarose gel electrophoresis. The DNA samples were stored in a refrigerator at −80 °C pending further processing.

### 2.4. Illumina-Based 16S rRNA Sequencing and Sequence Data analysis

The V3-V4 region of the bacterial 16S ribosomal RNA gene was amplified using the universal primers 338F (5′-ACT CCT ACG GGA GGC AGC AG-3′) and 806R (5′-GGA CTA CHV GGG TWT CTA AT-3′) by a GeneAmp 9700 thermocycler (ABI, New York, NY, USA). The PCRs were performed in triplicate: 20 μL of a mixture containing 2 μL of 10× PCR KOD-Plus-Neo buffer (Toyobo, Japan), 0.4 μL of KOD-Plus-Neo polymerase (Toyobo, Osaka, Japan), 2 μL of 2 mM dNTPs, 1.2 μL of 5 μM each primer, 1.2 μL of 25 mM MgSO_4_, 0.2 μL of 20 mg ml^−1^ BSA, and 10 ng of template DNA. The PCRs were conducted using the following program: 2 min of denaturation at 94 °C, 27 cycles of 5 s at 98 °C, 30 s at 55 °C, 30 s at 68 °C, and extension at 68 °C for 7 min. The amplicons were extracted from 2% agarose gels, purified using an AxyPrep DNA Gel Extraction Kit (Axygen Biosciences, Union City, CA, USA), and quantified using a QuantiFluor^TM^-ST fluorometer (Promega, Madison, WI, USA). The purified amplicons were pooled in equimolar concentrations and were paired-end sequenced (2 × 300) on an Illumina MiSeq platform (Majorbio Inc., Shanghai, China) according to standard protocols [24].

The paired-end reads were merged using Fast Length Adjustment of SHort reads (FLASH) software [25]. Quality filtering was performed using QIIME [26] with the following criteria: (i) the reads were truncated at any site that received an average quality score < 20 and any truncated reads that were shorter than 50 bp were discarded, (ii) sequences that overlapped by more than 10 bp were assembled according to their overlap sequence, and (iii) two nucleotide mismatches in primer matching and reads that contained ambiguous characters were removed. The operational taxonomic units (OTUs) were clustered with a 97% similarity cut-off using UPARSE and the chimeric sequences were identified and removed using UCHIME [27]. The most abundant sequence for each OTU was selected as the representative sequence, and the phylogenetic affiliation of each representative sequence was analyzed by the Ribosomal Database Project (RDP) Classifier (http://rdp.cme.msu.edu/, 15 July 2021) against the Silva128 16S rRNA database (http://www.arb-silva.de, 15 July 2021), with a 70% confidence threshold [28]. The alpha diversity was estimated using the ACE, Chao1, and Shannon indices that were calculated in Mothur. The shifts in bacterial community composition were visualized using principal coordinate analysis (PCoA) of the pairwise Bray-Curtis dissimilarity matrices based on a 97% OTU similarity across different samples. The linear discriminant analysis (LDA) effect size method (LEfSe, http://huttenhower.sph.harvard.edu/galaxy/, 2 November 2021) was performed for “biomarker” identification between different samples. Statistical analyses and the visualization of results were done in R 4.1.2. Normal distributions of the data were checked with the Shapiro–Wilkes test and homoscedasticity of variances was analyzed by the Leneve test. Significant differences in the parameter variances were evaluated, depending on the distribution of the estimated parameters, either with ANOVA or the Kruskal–Wallis rank sum test. Post hoc comparisons were conducted either by Tukey’s honest significant differences tests or pairwise Wilcoxon rank sum tests.

## 3. Results

### 3.1. Effects of CP and Biochar on Plant Growth

**Figure 1:**
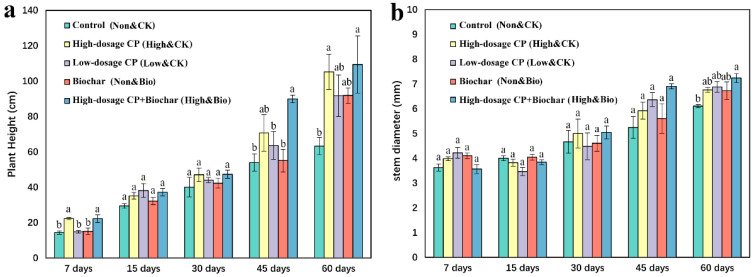
The heights (**a**) and stem thicknesses (**b**) of potted cucumbers with different treatments. Means (*n* = 3) with the same sampling date accompanied by the same lowercase letter were not statistically different (*p* ≥ 0.05) following the application of Tukey’s tests.

In the 7-day, 45-day, and 60-day samples, the heights of the cucumber seedlings in the soils that were treated with high-dosage CP were significantly higher than those in the control (Figure 1). In the 60-day samples, the stems of the cucumber seedlings in the soil treated with high-dosage CP fumigation and biochar amendments were significantly thicker than those of the control. The differences between the various treatments and the control group did not reach statistically significant levels (*p* > 0.05) in the other periods.

### 3.2. Effects of CP Fumigation and Biochar Addition on Rhizobacterial Alpha Diversity

Across all samples, a total of 2,880,091 raw sequence reads were successfully obtained from the 16S rRNA amplicon sequencing. After quality trimming, 2,050,800 sequences were retained, and the average length of the effective reads was 440 bp. Based on a 97% similarity level, a total of 6357 OTUs were obtained across all samples, and ~99.9% were classified as bacteria, with 43 phyla, 115 classes, 232 orders, 452 families, and 912 genera.

**Figure 2:**
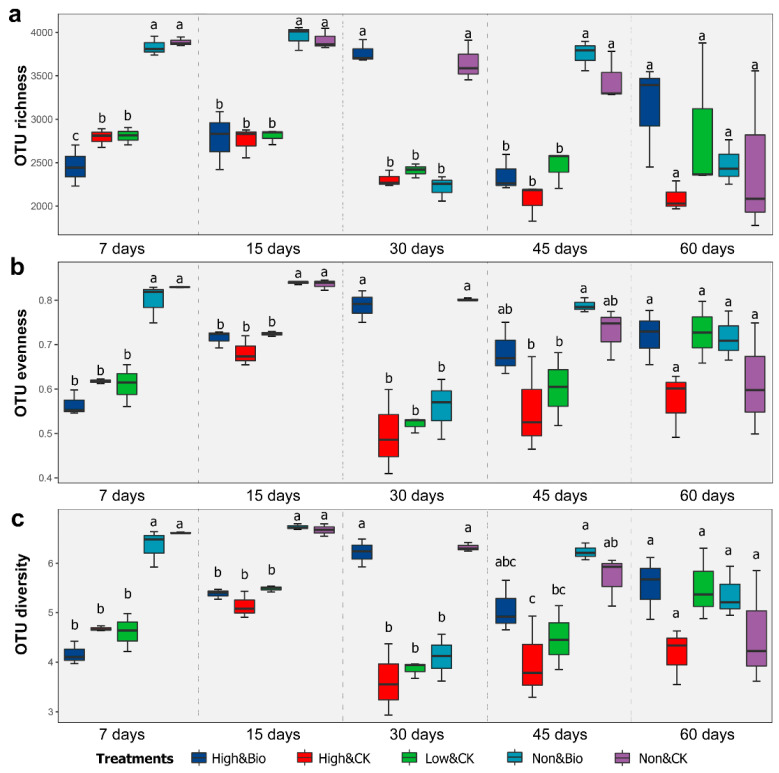
Alpha diversity estimates of the rhizobacterial communities. Box plots display the first (25%) and third (75%) quartiles, the median, and the maximum and minimum observed values within each data set. From top to bottom, the figure shows OTU richness estimates using the chao1 index (**a**), OTU evenness estimates using the Pielou index (**b**), and OTU diversity using the Shannon index (**c**). Means (*n* = 3) with the same sampling date accompanied by the same lowercase letter were not statistically different (*p* ≥ 0.05) following the application of Tukey’s tests.

As shown in Figure 2, both the high- and low-dosage CP treatments resulted in significant decreases (*p* < 0.001) in the rhizobacterial alpha diversity, including the richness, evenness, and diversity indices, compared to the control. Low-dosage CP showed a comparable inhibitory effect on the diversity, compared with high-dosage CP. At the late stage of cultivation, the rhizobacterial diversity gap between the CP fumigation and control treatments narrowed gradually, among which there were no significant differences among different treatments in the 60-day samples. The addition of biochar after CP fumigation significantly promoted the restoration of rhizobacterial diversity from the middle stage of cultivation. After 30 days of cultivation, there was no significant difference in diversity between the High & Bio and Non & CK treatments (except for the OTU richness of the 45-day samples).

### 3.3. Effects of CP Fumigation and Biochar Addition on the Structural Composition of Rhizobacterial Communities

**Figure 3:**
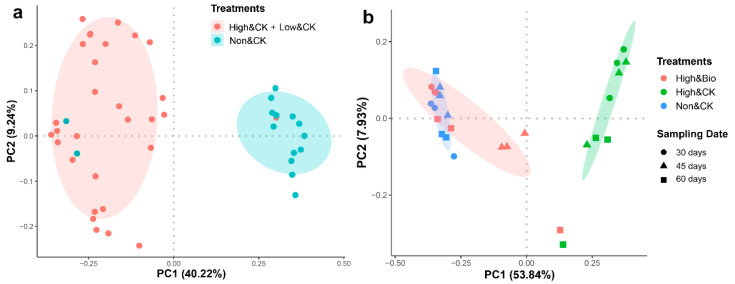
Principal coordinate analysis (PCoA) with the Bray–Curtis distances on the OTU level and displays differences in rhizobacterial communities. (**a**): includes samples with high- and low-dosage CP fumigation and unfumigation. Ellipses cover 68% of the data for each group. (**b**): includes 30-, 45-, and 60-day samples with high-dosage CP fumigation, biochar amendments, and unfumigation.

The PCoA revealed that the treatments were the determinants of the structural composition of the rhizobacterial communities (Figure 3). At the OTU level, the samples show a strong clustering of the rhizosphere bacterial communities according to CP fumigation in the first axis (Figure 3a). PC1 explained 40.22% and PC2 explained 9.24% of the total variations. The biochar additions resulted in changes in the structural composition of the rhizobacterial communities. The sample with biochar additions did not cluster completely according to their respective fumigation treatments, and the effect of biochar was mainly reflected in the middle and late stages of cultivation. To further identify the differences between the rhizobacterial communities in the biochar-amended soil and the control group, we used PCoA to analyze 30-, 45-, and 60-day samples with high-dosage CP fumigation, biochar amendments, and unfumigation (Figure 3b). PC1 explained 53.84% and PC2 explained 7.93% of the total variations. The High & Bio samples and High & CK samples are significantly separated in the first axis. The High & Bio samples clustered with the Non & CK samples (blue and red ellipses). To statistically support the visual clustering of the rhizobacterial communities in the PCoA, different treatments were examined using ANOSIM (analysis of similarities) based on the Bray–Curtis distances (Table S1). The results of ANOSIM were in agreement with the graphical PCoA display in Figure 3.

**Figure 4:**
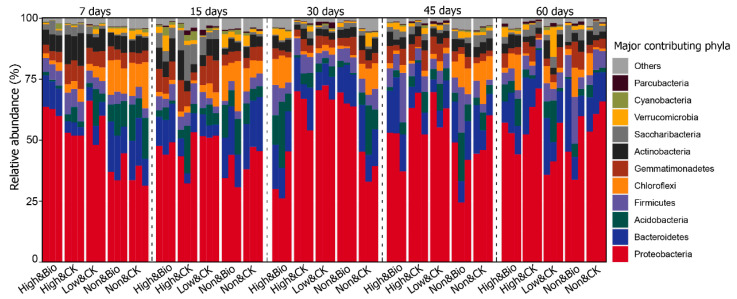
Bar plot of relative abundance of rhizobacterial communities in different samples on phylum level. From left to right, the figure shows the rhizobacterial composition of samples at 7, 15, 30, 45, and 60 days.

The structural composition of the rhizobacterial communities at the phylum level in different samples is shown in Figure 4. The predominant phyla were Proteobacteria, Bacteroidetes, Acidobacteria, Firmicutes, Chloroflexi, Gemmatimonadetes, and Actinobacteria, which accounted for >90% of the total rhizobacterial communities. The relative abundance range of Proteobacteria in all samples was 24.40~72.42%. To further investigate the most dramatic changes in the rhizosphere bacterial communities in the short term following CP application, we examined the differences in the relative abundance of the 7-day sample at the phylum level with ANOVA (Figure S3). The results showed that CP significantly increased the relative abundances of Proteobacteria, Gemmatimonadetes, Actinobacteria, Firmicutes, and Saccharibacteria in the early cultivation stage. Meanwhile, the relative abundances of Bacteroidetes, Chloroflexi, Verrucomicrobia, Planctomycetes, and Nitrospirae were significantly inhibited. It is worth noting that both the inhibition and promotion effects were temporary, as the differences in the rhizobacterial communities in terms of their abundances between the CP fumigated soil and control gradually diminished over time, with no significant differences from the control observed for the 60-day samples. Moreover, there were no significant differences in the composition of the major bacterial taxa between the Non & CK and Non & Bio treatments in the short term. Combined with the Bray–Curtis distances of samples from different treatments, the biochar addition after CP fumigation resulted in a significant difference from samples treated by CP alone at the phylum level (High & Bio vs. High & CK, *p* = 0.018, Table S1), and narrowed the differences between fumigated samples and control (High & Bio vs. Non & CK, *p* = 0.102, Table S1; Figure S1b), especially in the middle and late stages of cultivation.

### 3.4. Shifts in the Predominant Rhizobacterial Populations Following CP Fumigation and Biochar Addition

The changes in the abundances of the predominant species of the rhizobacterial communities in different samples are depicted in a heatmap (Figure S2). The species belonging to Proteobacteria accounted for more than half of all listed OTUs. Following CP fumigation, some genera were significantly stimulated, such as *Pseudomonas*, *Bacillus*, *Stenotrophomonas*, *Micromonospora*, and *Dongia*, whereas some taxa were significantly inhibited at different cultivation stages, such as *Acinetobacter*, *Dyadobacter,* Rhodospirillaceae, and *Pontibacter*.

**Figure 5:**
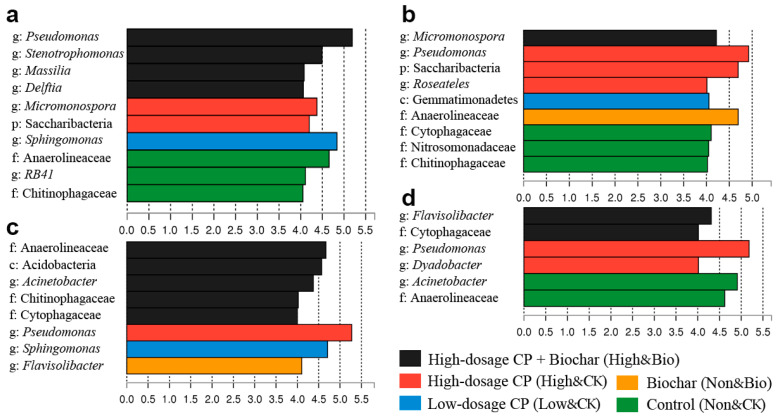
Linear discriminant analysis coupled with effect size measurements identifies the differentially abundant species between different samples. Figures (**a**–**d**) are the analyses of 7-day, 15-day, 30-day, and 45-day samples, respectively. Species with an LDA score higher than 4 are displayed.

The biomarkers that were identified by LEfSe (Figure 5) were highly consistent with the results shown in the heatmap. CP significantly affected the structural composition of the rhizobacterial communities. The abundances of some species increased significantly, such as those of *Pseudomonas*, *Micromonospora*, Saccharibacteria, *Roseateles*, and *Dyadobacter*. Moreover, *Sphingomonas* and Gemmatimonadetes were identified as biomarkers in the samples that were treated with low-dosage CP. However, the biochar additions to unfumigated soil had little impact on the predominant members of the rhizobacterial communities. Only Anaerolineaceae and *Flavisolibacter* were found through LEfSe in the 30-day and 45-day samples, respectively. Importantly, biochar amendments of the soils treated with CP significantly affected the composition of the predominant flora, with more biomarkers found at different cultivation phases, including *Pseudomonas*, *Stenotrophomonas*, *Massilia*, *Delftia*, *Micromonospora*, Anaerolineaceae, Acidobacteria, *Acinetobacter*, Chitinophagaceae, Cytophagaceae, and *Flavisolibacter*. Among the bacteria that were stimulated by biochar amendments, some taxa, such as *Bacillus*, *Massilia*, and Chitinophagaceae, have been reported to be beneficial for the degradation of pollutants and to also protect plants by producing antifungal effectors to suppress plant pathogens (Table S2). Furthermore, LEfSe did not show any significantly different genera at a threshold of 4 in the 60-day samples, which indicated that the differences in the rhizobacterial communities among different treatments gradually narrowed over time.

## 4. Discussion

### 4.1. Effects of CP and Biochar on the Growth of Cucumber Seedlings

Previous studies have reported that while effectively preventing soil-borne diseases, CP promoted the growth and yield of crops [4,29]. In agricultural production, fumigants are often used on crops with high economic value and especially for severe occurrences of soil-borne diseases [4]. Therefore, the “fertilizer effect” of fumigants is probably caused by the elimination of pathogenic bacteria that infect plants, but this has not been widely confirmed. Similarly, although research on biochar additions to soil has seen a surge and our knowledge has significantly advanced over the past decade, the effects of biochar additions on crop growth still seem to be unpredictable [30]. In our study, the biochar additions after CP fumigation led to the highest physiological indicator values at 45 d and 60 d, and the differences of plant heights at 45 d and 60 d and stem diameters at 60 d from the control reached significant levels (*p* < 0.05).

In other studies, the magnitude of crop response varied with biochar characteristic [31], application rate [32], soil type [33], plant species [33], and other artificial input factors. The response of cucumber seedlings observed in this study is similar to the previous study by Van Zwieten et al. [33], who found that there was an interaction between biochar and fertilizer addition, and plants had little response to biochar in the absence of fertilizer. In our study, biochar addition after CP fumigation led to the significant growth of a large number of beneficial microorganisms (Table S2 and Figure 5) in the rhizosphere bacterial community, which could produce the potential agronomic benefits on plants. Most of these beneficial taxa were not recorded in the separate biochar-amended soil without CP fumigation. The empty niche caused by CP fumigation is likely to create the necessary preconditions for biochar to stimulate beneficial populations. This may just be the reason why the physiological indicators of cucumber seedlings in biochar-amended soils after CP fumigation increased significantly. However, further field studies need to be conducted to verify the hypothesis that changes in plant physiological indicators are attributable to the combined effects of CP and biochar.

### 4.2. Different Changes between the Bulk Soil Bacterial Communities and Rhizosphere Soil Bacterial Communities after CP Fumigation

The effects of CP on the microbial communities appeared mainly in the early and mid-stages of the cultivation experiments, which should be related to the short half-life of CP in the soil [21]. The top members of the rhizobacterial communities were mainly the same as previously reported for bulk soil bacterial flora, such as Proteobacteria, Firmicutes, and Acidobacteria [7,20]. The difference was that Bacteroides and Chloroflexus were more abundant in the rhizobacterial communities (Figure 4). Moreover, some phyla that changed significantly in the bulk soil bacterial communities also exhibited similar change features in the rhizosphere soil bacterial flora, including a decrease in the relative abundance of Verrucomicrobia, Chloroflexi, and Planctomycetes and an increase in the relative abundance of Firmicutes. However, inconsistencies and even opposite patterns were observed for the changes in phylum abundances. For example, the relative abundances of Proteobacteria and Actinobacteria increased significantly in the rhizobacterial communities, but did not change significantly or even decreased in the bulk soil bacterial communities [7,20]. Studies have shown that Proteobacteria and Actinobacteria harbor genera and species with activity against plant pathogenic fungi [11].

CP increased the relative abundances of some functional genera in the rhizobacterial communities. *Pseudomonas* and *Stenotrophomonas* harbor biocontrol strains that can inhibit many economically critical fungal pathogens in the soil, such as *Fusarium oxysporum*, *Gaeumannomyces graminis,* and *Rhizoctonia solani* [11,34,35]. In addition to *Pseudomonas*, we also found several other predominant populations that are associated with pollutant biodegradation, including *Stenotrophomonas*, *Dongia*, *Sphingomonas*, and *Roseateles*. These taxa-harboring species have been isolated from various environments that were contaminated by organic compounds [36,37]. In addition, the changes in the abundances of rhizosphere functional bacterial genera, including *Bacillus*, *Pseudomonas, and Sphingomonas’* increase and *Acinetobacter’s* decrease found in this study were consistent with previous studies on the bacterial flora in bulk soils after CP fumigation [7].

In this study, *Nitrospira* and *Nitrosospira* were found to be biomarkers in the control samples (the LDA values were 3.4 and 3.0, respectively). *Nitrospira* and *Nitrosospira* are essential nitrite-oxidizing bacteria (NOB) and ammonia-oxidizing bacteria (AOB) in the soil. This finding not only indicated that the two genera are predominant nitrifiers in the rhizosphere soil but also suggested that the CP treatments inhibited their relative abundances. This inhibition effect would limit ammonia oxidation and the nitrification processes that are involved in nitrogen cycling in rhizosphere soils. This result is consistent with previous studies. Fang et al. [20] found that CP fumigation significantly increased NH_4_^+^-N concentration and decreased NO_3_^−^-N concentration in the early stage, and the abundance of *Nitrospira* in the bulk soil was inhibited by CP. Yan et al. [29,38] found that CP inhibited the nitrification process and stimulated the denitrification process in the bulk soil, which may change the flux of the agricultural greenhouse gas, N_2_O, from the soil. The changes in the relative abundances of the nitrifiers in response to CP also agreed with the results of [39], who found that CP significantly inhibited the abundance of the ammonia monooxygenase-encoding gene (*amoA*) in AOB.

### 4.3. Biochar Accelerated the Reconstruction of Rhizobacterial Communities after CP Fumigation

The alpha diversity analysis (Figure 2) showed that there were no significant differences between the rhizobacterial diversity of the Non & Bio treatment and the Non & CK treatment. This result suggested that biochar in the soil without CP fumigation did not stimulate rhizobacterial diversity. However, for the soil fumigated by CP, the rhizobacterial diversity of the biochar treatments recovered more quickly and reached the unfumigated level at 30 d compared to the control. The results indicate that biochar additions benefit the restoration of rhizobacterial diversity and shorten the time for the diversity to recover to a level similar to that of the control, when microbial abundances are at a low level.

The observed positive response could be partly explained by the high carbon and ash content of biochar (the C content of biochar used in this study is 68%, and the ash content is 4.5%). This bio-available carbon and minerals in ash are valuable resources in the soil food web, including some essential macro- and micro-nutrients required by microorganisms, which can promote the rapid restoration of microbial population diversity [15]. The biochar produced by *Ageratina adenophora* in this study has a high specific surface area of 162.18 m^−2^·g^−1^, which may create a more favorable and separate pore habitat in the root zone and benefit the restoration of microorganisms [40]. Previous studies in our laboratory have shown that the characteristics of biochar can accelerate the release of the inhibitory effect of fumigants on microorganisms [16,41]. The finding that biochar can accelerate the recovery of rhizobacterial diversity is similar to the study in the bulk soil by Fang et al. [20], who found that 5% biochar amendment could counteract the inhibitory effect on bulk soil bacterial diversity. However, the reasons for the accelerated recovery of diversity may vary. For example, compared with the high biochar application rate (5%) in the study by Fang et al., the 2% biochar addition rate in this study is only at a medium level [42]. Previous studies have shown that the addition of biochar before soil fumigation can accelerate the degradation of the fumigant and may lead to a decrease in fumigation efficacy, resulting in the rapid release of microbial inhibition [16,17]. In addition, rhizobacterial diversity had a brief peak at 30 d, especially in OTU richness, which may be susceptible to the nutrients provided by biochar. But these nutrients may not be sufficient to support the high richness of communities for a long time. This may be the reason for the decline in diversity at 45 d. The diversity data at 60 d have a large degree of dispersion and show characteristics that are not exactly the same as before, which may be closely related to soil aging in the later stage of the experiment.

### 4.4. New Predominant Rhizobacterial Populations after Biochar Amendments

There may be a temporary biological vacuum in microbiome niches after fumigant applications [43]. In our study, the decrease in rhizobacterial diversity was consistent with the niche vacuum phenomenon. Biochar amendments reduced the difference between fumigated samples and unfumigated samples (Figure 3 and Figure S1). Nevertheless, it is worth noting that the community composition of biochar-amended samples is not completely consistent with the original samples. This may be closely related to the rapid colonization of populations stimulated by biochar in the empty niches after CP fumigation (Figure 5 and Figure S2).

These biomarkers that were identified in biochar-amended samples have been reported to be related to a wide variety of potential biotechnological applications, such as plant growth promotion, bioremediation, and bioconversion of pollutants (Table S2). Some of them, such as *Acinetobacter*, *Delftia*, and *Micromonospora*, have plant-growth-promoting traits including nitrogen fixation, siderophore production, and mineral solubilization. *Bacillus*, *Massilia*, Chitinophagaceae, and Flavobacteriaceae have been found to possess remarkable biocontrol abilities that suppress diseases that are caused by plant pathogens and can protect plants by producing antifungal effectors or by stimulating host defense mechanisms. *Pseudomonas* and *Acinetobacter* harbor essential denitrifiers in the rhizosphere. Moreover, many bacteria belonging to these taxa have also been reported to degrade a variety of soil pollutants, such as aniline, polycyclic aromatic hydrocarbons, herbicides, and so on.

The increases in the relative abundances of these bacterial taxonomic groups that were stimulated by biochar additions will have a potentially beneficial impact on the rhizobacterial functional diversities and plant growth. Notably, biochar amendments of soils without CP fumigation exhibited no such change characteristics and did not result in as many new predominant members that were related to beneficial functions. Therefore, we further believe that the large number of empty microbiome niches that were caused by CP created an essential prerequisite for biochar to induce the transplantation of beneficial populations in empty niches and to become top members of the bacterial communities in the rhizosphere.

The results caused by biochar are closely related to its characteristics. The biochar used in this study has a pH of 10.5 and has an ash content (rich in plant-available basic cations) of 4.5%. These factors may significantly change the rhizosphere soil environment, including its acidity [33]. In addition, the high SSA and microporosity of biochar may create favorable conditions for the colonization of new populations. For example, microorganisms could avoid space competition by exploring pore habitats in biochar [15]. The adsorption of essential nutrients on biochar allows some populations to colonize and meet their mineral nutrient needs. However, the recalcitrance of biochar suggests biochar probably have selectivity, and part of bacterial populations may not be able to meet their carbon needs from biochar and thus are not encouraged to colonize [40]. Therefore, the change of root zone environment, in conjunction with a reduction in soil acidity and the selectivity of biochar, is probably responsible for the change of the predominant members of the rhizobacterial communities in biochar-amended soils following CP fumigation.

## 5. Conclusions

This is the first study that reports the responses of rhizosphere soil microbial communities to biochar amendments after CP fumigation. CP significantly inhibited rhizobacterial diversity and shifted the structural composition of the rhizobacterial communities. CP significantly increased the relative abundances of Proteobacteria, Gemmatimonadetes, Actinobacteria, Firmicutes, and Saccharibacteria, and decreased the relative abundances of Bacteroidetes, Chloroflexi, Verrucomicrobia, Planctomycetes, and Nitrospirae. CP had a strong inhibitory effect on essential nitrifying genera, including *Nitrospira* and *Nitrosospira*. Biochar amendments after CP fumigation shortened the time for the taxonomic diversities to recover to their unfumigated levels. The relative abundances of many taxa related to plant growth promotion and pollutant bioremediation increased, including *Pseudomonas*, *Stenotrophomonas*, *Bacillus*, *Massilia*, *Acinetobacter*, *Delftia*, *Micromonospora*, Cytophagaceae, and *Flavisolibacter*. These changes, which were stimulated by biochar amendments, would promote bacterial functional diversity in the rhizosphere and benefit plant growth and disease resistance. Future studies should target the isolation and cultivation of these beneficial microorganisms, as well as their application in sustainable crop production.

## Data Availability

The data that support the findings of this study are available from the corresponding author, A.L., upon reasonable request.

## Supplementary Materials

The following supporting information can be downloaded at: https://www.mdpi.com/article/10.3390/ijerph19042126/s1: Figure S1: Sample distance comparisons based on Principal coordinate analysis (PCoA) with Bray–Curtis distances; Figure S2: The heatmap summarizes the composition of rhizobacterial communities over time; Figure S3: Relative abundance differences of rhizobacterial communities in 7-day samples at phylum levels; Table S1: Differences of rhizobacterial community structures between treatments are calculated using ANOSIM based on the Bray-Curtis distances at two phylogenetic levels: the phylum level and the OTU level (999 permutations); Table S2: Bacterial functional taxa stimulated by biochar amendments and their roles in the rhizosphere soil.
