# Supplementary material for: Impact of Biochar on Rhizosphere Bacterial Diversity Restoration Following Chloropicrin Fumigation of Planted Soil
Source: Int J Environ Res Public Health. 2022 Feb 14;19(4):2126. doi: 10.3390/ijerph19042126 (PMC8872450; doi:10.3390/ijerph19042126)
Supplement: Supplementary file 1 [file ijerph-19-02126-s001.zip › ijerph-1576437-supplementary.pdf]

Supplementary Materials

for

Impact of biochar on rhizosphere bacterial diversity restoration following chloropicrin fumigation of planted soil

Jun Li[1, 2], Yan Chen[3], Xiangyang Qin[1], Aocheng Cao[2], and Anxiang Lu[1, *]

1. Institute of Quality Standard and Testing Technology, Beijing Academy of Agriculture and Forestry Science, Beijing, 100097, China

2. Institute of plant protection, Chinese Academy of Agricultural Sciences, Beijing, 100193, China

3. COFCO Nutrition & Health Research Institute, Beijing, 102209, China

[*]Corresponding author: luax@brcast.org.cn; Phone: +86 10-51503057; Fax: +86 10-51503793

Table S1 Differences of rhizobacterial community structures between treatments are calculated using ANOSIM based on the Bray-Curtis distances at two phylogenetic levels: the phylum level and the OTU level (999 permutations). Significance levels: *$P \leq 0.05$; **$P \leq 0.01$; ***$P \leq 0.001$. R, ANOSIM test statistic. CP fumigation group includes High&CK and Low&CK treatments, unfumigation group includes Non&CK treatment, and Biochar addition group includes Non&Bio and High&Bio treatments.

**Phylogenetic level**	**Phylum**		**OTU**	
**ANOSIM output**	***R***	***P***	***R***	***P***
CP fumigation vs Unfumigation	0.3651	0.001***	0.7036	0.001***
CP fumigation vs Biochar addition	0.1748	0.001***	0.267	0.001***
Biochar addition vs Unfumigation	-0.0592	0.894	0.0574	0.086
High&CK vs High&Bio	0.0979	0.018*	0.2345	0.001***
High&Bio vs Non&CK	0.0621	0.102	0.2184	0.009**

Table S2 Bacterial functional taxa stimulated by biochar amendments and their roles in the rhizosphere soil

Taxa	Phyla	Role
Pseudomonas	Proteobacteria	Inhibit plant pathogens<br>Promote plant growth<br>Biodegrade pollutants<br>Denitrifying<br>A low number of plants pathogenic species
Stenotrophomonas	Proteobacteria	Inhibit plant pathogens<br>Biodegrade pollutants<br>Promote plant growth
Bacillus	Firmicutes	Inhibit plant pathogens<br>Promote plant growth
Massilia	Proteobacteria	Promote plant growth<br>Biodegrade pollutants<br>Nitrogen-fixing
Acinetobacter	Proteobacteria	Inhibit plant pathogens<br>Nitrogen-fixing<br>Denitrifying
Delftia	Proteobacteria	Inhibit plant pathogens<br>Nitrogen-fixing
Micromonospora	Actinobacteria	Inhibit plant pathogens<br>Nitrogen-fixing
Chitinophagaceae	Bacteroidetes	Protect plants by producing antifungal effectors to suppress plant pathogens
Flavobacteriaceae	Bacteroidetes	Protect plants by producing antifungal effectors to suppress plant pathogens

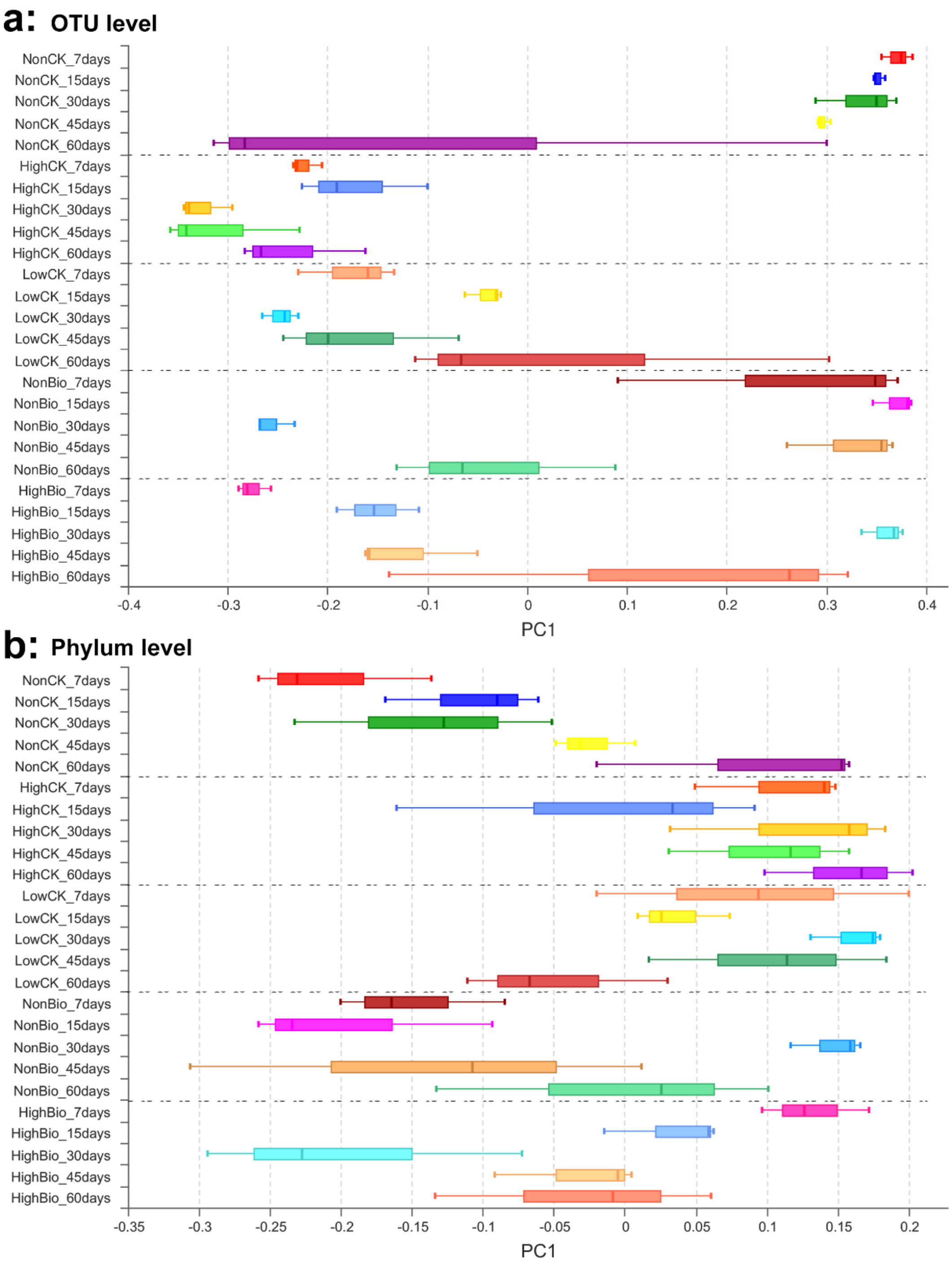

Figure S1 Sample distance comparisons based on Principal coordinate analysis (PCoA) with Bray–Curtis distances. The box plots represent the dispersion of different groups of samples on the PC1 axis. a: OTU level; b: phylum level.

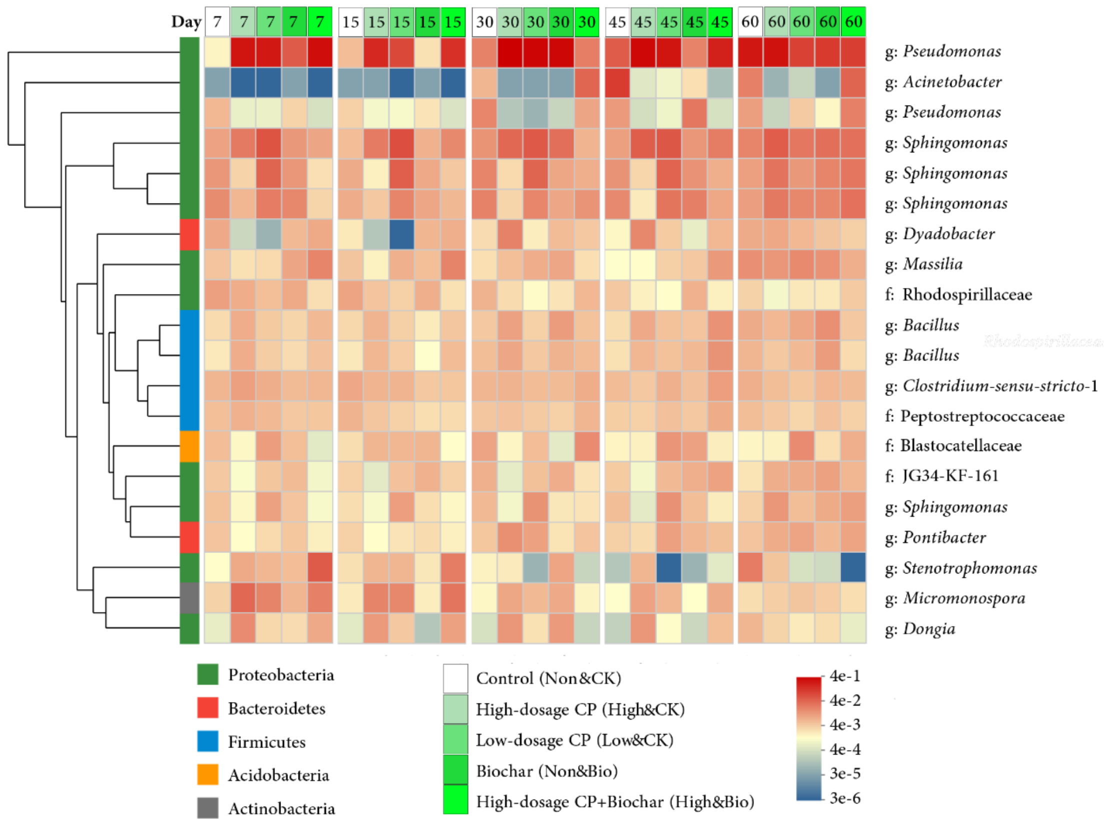

Figure S2 The heatmap summarizes the composition of rhizobacterial communities over time. The cell values represent normalized relative abundance, which are the mean values of five repeated samples. The top 20 abundant OTUs are listed, and the species (genus and family, denoted as g and f, respectively) corresponding to each OTU are listed on the right. Different treatments are represented as varying degrees of green and are shown in grids at the top of the heatmap. The numbers in the grid indicate the sampling time, with the 7, 15, 30, 45, and 60-day samples from left to right.

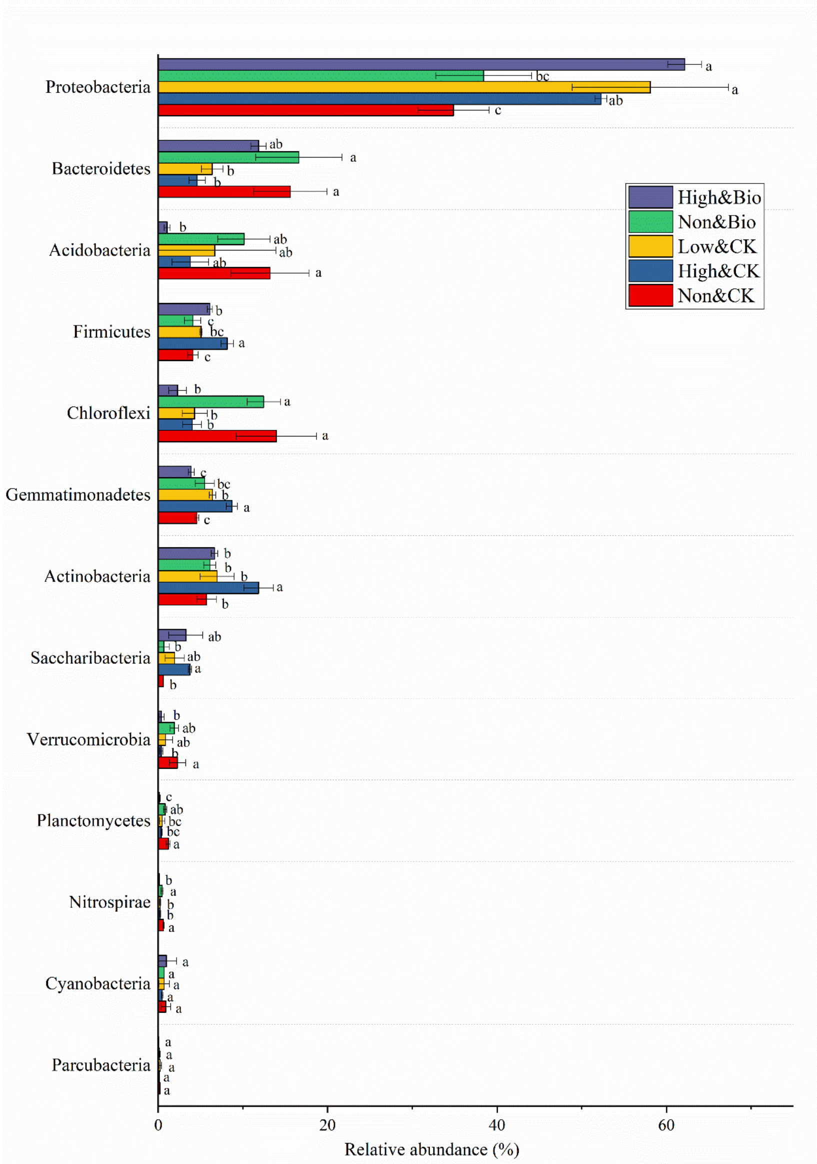

Figure S3 Relative abundance differences of rhizobacterial communities in 7-day samples at phylum levels. Means (n=3) with the same lowercase letter were not statistically different ($P \geq 0.05$) following the application of Tukey's tests.
